# Robust and Secure Data Transmission Using Artificial Intelligence Techniques in Ad-Hoc Networks

**DOI:** 10.3390/s22010251

**Published:** 2021-12-30

**Authors:** Pooja Rani, Kavita, Sahil Verma, Navneet Kaur, Marcin Wozniak, Jana Shafi, Muhammad Fazal Ijaz

**Affiliations:** 1 School of Computer Science and Engineering, Rayat Bahra University, Mohali 140413, India; pooja.rani@rayatbahrauniversity.edu.in; 2 Department of Computer Science and Engineering, Chandigarh University, Mohali 140413, India; kavita@ieee.org (K.); sahilverma@ieee.org (S.V.); navneet.e11384@cumail.in (N.K.); 3 Machine Learning and Data Science Research Lab, Chandigarh University, Mohali 140413, India; 4 Bio and Health Informatics Research Lab, Chandigarh University, Mohali 140413, India; 5 Bio-Intelligence Research Lab, Chandigarh University, Mohali 140413, India; 6 Faculty of Applied Mathematics, Silesian University of Technology, 44-100 Gliwice, Poland; 7 Department of Computer Science, College of Arts and Science, Prince Sattam Bin Abdul Aziz University, Wadi Ad-Dawasir 11991, Saudi Arabia; j.jana@psau.edu.sa; 8 Department of Intelligent Mechatronics Engineering, Sejong University, Seoul 05006, Korea

**Keywords:** MANET, AODV, Black Hole Attack (BHA), Artificial Bee Colony algorithm, artificial neural network

## Abstract

The paper presents a new security aspect for a Mobile Ad-Hoc Network (MANET)-based IoT model using the concept of artificial intelligence. The Black Hole Attack (BHA) is considered one of the most affecting threats in the MANET in which the attacker node drops the entire data traffic and hence degrades the network performance. Therefore, it necessitates the designing of an algorithm that can protect the network from the BHA node. This article introduces Ad-hoc On-Demand Distance Vector (AODV), a new updated routing protocol that combines the advantages of the Artificial Bee Colony (ABC), Artificial Neural Network (ANN), and Support Vector Machine (SVM) techniques. The combination of the SVM with ANN is the novelty of the proposed model that helps to identify the attackers within the discovered route using the AODV routing mechanism. Here, the model is trained using ANN but the selection of training data is performed using the ABC fitness function followed by SVM. The role of ABC is to provide a better route for data transmission between the source and the destination node. The optimized route, suggested by ABC, is then passed to the SVM model along with the node’s properties. Based on those properties ANN decides whether the node is a normal or an attacker node. The simulation analysis performed in MATLAB shows that the proposed work exhibits an improvement in terms of Packet Delivery Ratio (PDR), throughput, and delay. To validate the system efficiency, a comparative analysis is performed against the existing approaches such as Decision Tree and Random Forest that indicate that the utilization of the SVM with ANN is a beneficial step regarding the detection of BHA attackers in the MANET-based IoT networks.

## 1. Introduction

The Internet of Things (IoT) is an innovative technology that allows physical things such as homes, vehicles, hospitals, and many more to be integrated with the digital world through an internet connection [1]. The demand and the use of IoT techniques have grown significantly over the last couple of years. Using this technique, physical things are made smarter and have found application in smart buildings [2], transportation [3], healthcare systems [4], and many more. IoT gathers data from physical things, stores it and then communicates it to different networks. Here, we considered IoT in the Mobile Ad-Hoc Network (MANET).

**Figure 1:**
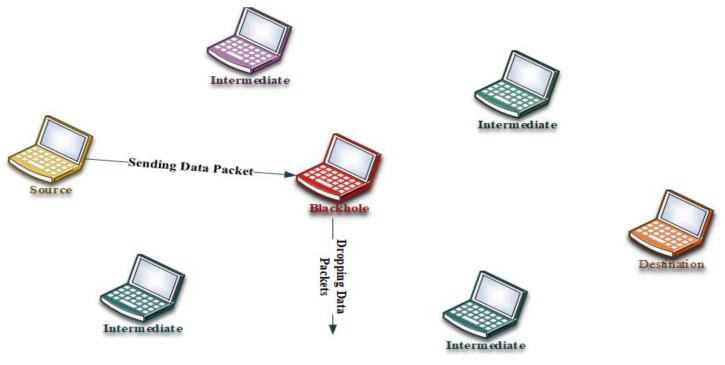
Packet drop by black hole node.

In MANET, the nodes are movable and any nodes are able to be processed as a host as well as a router within the network [5]. Therefore, MANET is more vulnerable to security attacks including active and passive attacks in the network. MANET comprises mobile/portable wireless devices that communicate without the requirements of fixed infrastructure. Since the nodes can be moved freely, the network topology is dynamic and can easily leave and join the network when required [6]. In MANET, nodes can easily communicate with different nodes that are lying in the range of communication. As shown in Figure 1, N3 and N2 are in the communication range of node N1. Therefore, N1 can forward its data to the N3 and N2 nodes directly [7]. These nodes are known as the neighbor nodes. The node that is not connected is directed to the communicating node (Source node) and a route is formed by other nearby nodes. The data transmission in MANET takes place in a safe and forward manner where each node acts as a router. The address of the destination node is discovered without any additional methods. Further, a distinguishing work in the field of intrusion detection was also presented by Panigrahi et al. [8,9].

Inside the MANET, every node is treated as a router as well as a host. A collection of rules, known as protocols, is established to move data between different nodes inside the network. The protocols in MANET may be classified into two types: proactive and reactive routing protocols [10], with proactive routing procedures deciding on route construction based on the features of the previously stored node d in the routing table, also known as table-driven routing protocols. In reactive routing, on the other hand, the creation of the route is done on demand [11]. For route generation in this study, we employed the Ad-hoc On-Demand Distance Vector (AODV) routing protocol. Section 3 provides a more extensive description.

The network has vulnerability to multiple Service Denial (DoS) attacks, such as black holes, gray holes, sinkholes, and many more. For this reason, the design and implementation of such a network are based on the following assumptions: inside the network, nodes are depending on each other, however some nodes are irresponsible. These undesired nodes degrade network performance whenever they get a chance. In the Black Hole (BHA) node, the complete data traffic is attracted by the malicious nodes by sending the highest hope count towards the source node. As per assumptions of the source node, the node with a higher sequence number is genuine and forwards the entire data [12].

Several researchers have used different techniques either individually or in combination with other approaches to minimize the effect of the Black Hole Attack (BHA) in MANET. These techniques include security IOT [13], sequence number of thresholds [14], acknowledgment [15], cross-checking [16], trust [17], cross-layer [18], and clustering [19], including others. The simulation was performed using the MATLAB simulation tool in which the detection of black hole node was performed using a modified AODV protocol, which is designed by integrating optimization with a Machine Learning (ML) approach.

## 2. Background

In BHA, the influenced node used its protocol of routing to discover the destination node itself, having the most limited route that contains the advanced information about the destination node. Despite checking its routing table, this dynamic node will reveal the accessibility of its new path. In this kind of threat, the affected node consistently has the availability to capture the routing information and modify the packet of data as well as discard them. In a flood-based protocol, the reply of the malicious node will be received by the requesting node before getting any kind of reply from any genuine node; in this way, affected and fake paths will be created. After setting up this path, it is presently up to the node whether to drop the packet or to forward the packet to an unidentified location [20]. A Route Request (RREQ) packet is sent to all other nodes through the sensor nodes [21]. A response message (RREP) is transferred back to the source node after receiving the REEQ packets in a special case if the node contains the acquired destination address. If not, the packet is sent to its nearest node, by which the data reach the destination side. The RREP message is sent back to the destination node via the same route after receiving the RREQ message. Therefore, the path is established between the source as well as the destination node. 

However, at the same time, the BHA node also sends RREP messages quickly as well as with a higher hop count compared to the node on the destination side [22]. This is the behavior of the source node to send data to the node having a higher hop count and then pass data up to the node of BHA. The dropping of data packets has been started and hence leads to a DoS attack inside the network; this scenario is presented in Figure 1.

The nodes’ behavior can be represented based on their hop count, which is listed in Table 1. The value of hop count “High” and “Low” represents the true and false information contained in the RREP packet [23].

In this research, an Artificial Bee Colony (ABC) algorithm is applied to select the best route (that consumes minimum energy) between a source node and destination node. An asymmetric review was presented by [24] that shows a survey on Swarm Intelligence (SI) techniques and states that it can be used to resolve the problem of feature selection. The concept of SI is increasingly intertwined with algorithms for processing and optimizing the large amount and flows of information. In [25], a survey on different SI algorithms along with their role in the IoT is presented. The authors stated that SI in a wireless network can be used for Cluster Head (CH) selection, for sensor deployment, node localization, and in IoT to minimize the number of hops from the sensor node to the sink node. In [26], Ant Colony Optimization is used as a SI approach in cloud computing to solve the problem of task scheduling. Application of SI in a Cyber-Physical System (CPS) along with challenges is presented by [27]. In MANET, there are several SI techniques available including Firefly, Ant Colony Optimization, Cuckoo Search, Particle Swarm Optimization, ABC, and many more. In this research, we used ABC as an SI approach to select the route formed by the AODV routing protocol. In this research, we used ABC as an SI approach to select the route formed by the AODV routing protocol.

Instead of other SI techniques, we have ABC as an optimization algorithm because:

- It is a fast searching algorithm
- It can make a decision very frequently about which node is capable of communicating with others by consuming minimum energy.
- To achieve its target ABC using the velocity of searching time bees, and to search the target, a number of employee bees can be used.

It is a quality-dependent searching algorithm that repeats its process until it delivers the best data per the requirement. The remainder of the paper is structured as follows: In Section 3, the literature on the black hole attack is discussed. Section 4 and Section 5 present the methodology and findings, respectively. In Section 6, the conclusion is offered, followed by references.

## 3. Related Work

Shahabi et al. [23] designed a novel routing algorithm in addition to AODV to secure a network from BHA. Using this strategy, the malicious nodes are identified based on the node’s behavior. If any are detected that node is deleted from the route. The experiments also show better Packet Delivery Rate (PDR) with reduced delay. Baadache and Belmehdi [24] presented an acknowledgment-based routing approach by which the communicating nodes send acknowledgment whenever the nodes receive the data packet. The algorithm suffers from high routing overhead as each node sends an acknowledgment message to the prior node. In addition to the above problem, Kumari and Paramasivan [25] developed a routing mechanism of trust where the behavior of nodes is analyzed based on the dropping rate of packets, but this protocol also suffers from high overhead because of the additional use of control packets. Gurung and Chauhan [26] used the approach of mitigating a Gray Hole Attack (GHA) that takes the help of other nearby nodes, known as the nodes of the Intrusion Detection System (IDS), to monitor the performance of other communicating nodes. In the appearance of any malicious node, the packet drop value of the node is higher. In this case, the important message (“ALERT”) is transferred among the networks to intimate other nodes to separate attacker nodes. As the algorithm works on the defined threshold, proper positioning of special nodes is required. Mohanapriya and Krishnamurthi [14] designed a new approach source node that imitates the destination node of the total amount of packets transmitted from all expected routes. Query request is transmitted by the destination node, particularly in the case where the node cannot obtain the desired packets. In response to this query reply, a message is sent back to the node that is about two-hop counts in contrast to the destination node. Once the message of query reply is received, the destination node compares its prior-received data with the recently received data. In case an error appears, consider that node as the suspected node and add it to the list of malicious nodes. Keerthika and Malarvizhi [27] presented a combined trust-based bee approach to secure the network against BHA. ABC is used for the detection of a secure route. A new solution is generated based on the fitness function of bees. The designed algorithm shows enhancement in the PDR and end-to-end delay. Merlin and Ravi [28] presented a new trust-based approach that works on energy-aware routing for MANET. The BHA has been detected for single as well as for multiple routes formed during the data communication process. Rezaei et al. [29] presented a mechanism in which the source node transmits the route response data packet after processing the node’s information, which is later used for BHA detection. Whether the node is genuine or malicious is decided by the intermediate node. On the other hand, Yasin et al. [30] used a timer and baiting-based method for BHA detection in MANET. Monica Sood et al. [31] used a deep learning model for traffic flow prediction based on attention for inventory automation using a Wireless Sensor Network.

## 4. Proposed Work

**Figure 2:**
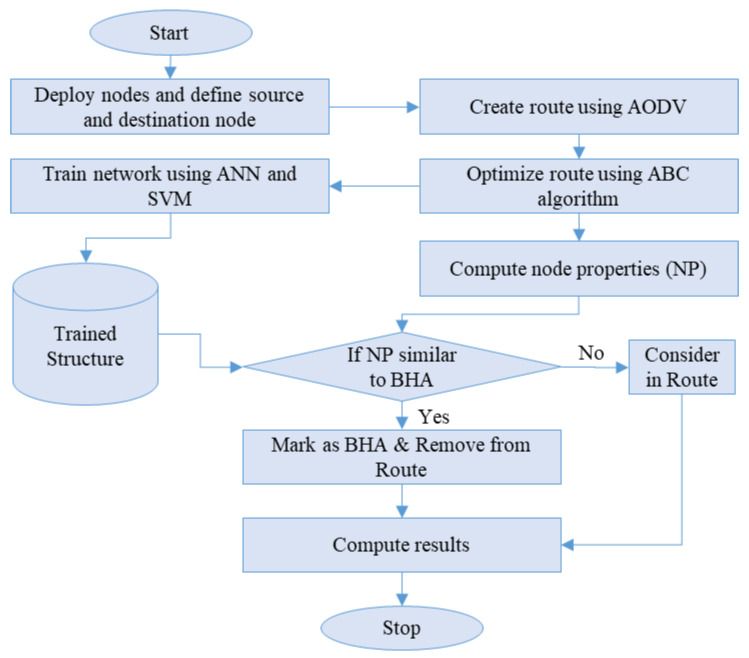
The flow of proposed work.

In this research, an enhanced routing protocol (i.e., AODV) is proposed and utilized in the detection of BHA nodes. The entire flow is provided in Figure 2. The proposed work is mainly partitioned among three sub-parts.

The route is first established using the AODV routing protocol, then optimized using the Artificial Bee Colony algorithm’s fitness function before classifying the nodes based on their attributes. Table 2 lists the parameters that were considered for the system.

### 4.1. Deploy Nodes and Define the Source and Destination Node

Initially, N number of nodes are deployed within a defined length and width of (1000 × 1000). Each node is labeled by (N_1_, N_2_, …, N_n_), including the source (N_16_) and the destination node (N_36_) where n is the number of deployed nodes.

### 4.2. Routing Mechanism

The route has been formed using AODV as a routing protocol, which establishes route on an on demand basis and hence reduces the number of required broadcasts. Using this protocol, the nodes that are not part of the route are not needed to manage the information of routing. Thus, it can be known as a pure on-demand basis process of routing. This reduces the routing packet size. During the route discovery process, two packets such as RREQ and RREP are responsible for forming the route. Both control messages contain an essential attribute known as ‘destination sequence number’.

**Figure 3:**
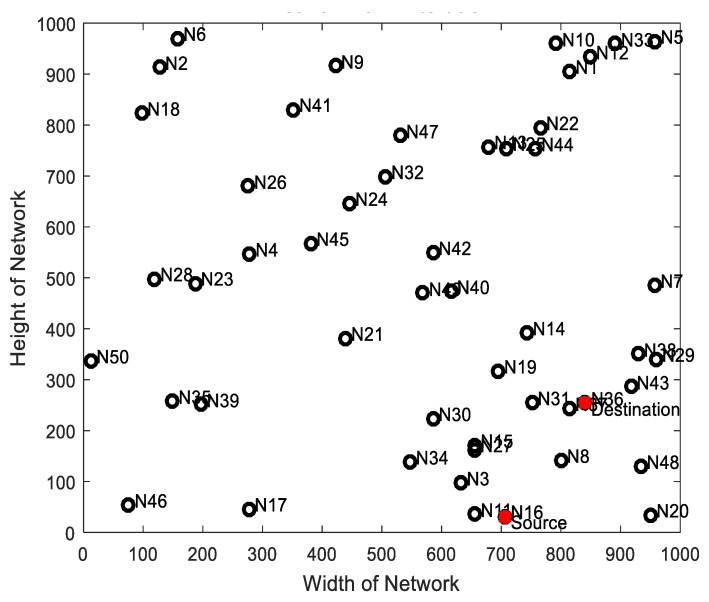
Node deployment.

The enhanced value of this number determines the most suitable path. As presented in Figure 3, the source node (S) broadcasts RREQ messages, which are received by nearby nodes denoted by N2, N6, and N3, respectively. 

This process is initiated to determine the destination node (D). After receiving their message, the nearby nodes (N2, N6, and N3) send back:

(i) IF the node is identified as a destination node then the RREP message is transferred back to the source node.
(ii) If not, then its routing table needs to be updated with fresh information on the path regarding the destination node.

After reaching the RREQ packet on destination (D), this process is stopped.

**Figure 4:**
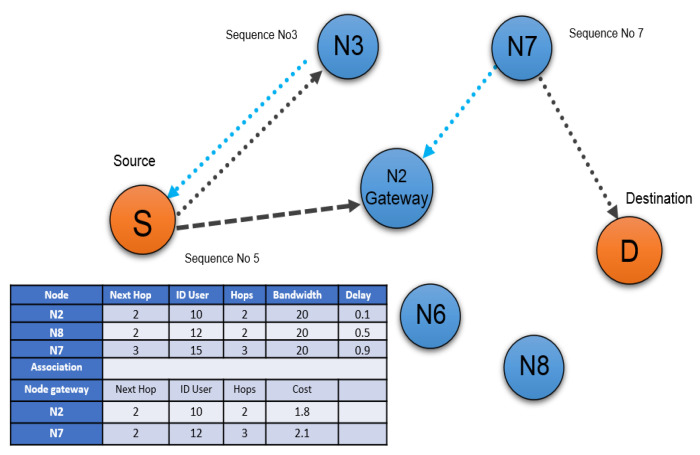
AODV routing Process.

Then the node checks the sequence number of destinations from the routing table. If this sequence number is higher compared to the sequence number of destination then the route has been created through that particular node as shown in Figure 4. Node N3 has a higher sequence number than N2 and N6. Therefore, the final route is created by (S, N3, N2, N7, and D). The algorithm followed for AODV (Algorithm 1) is provided below in pseudo-code.

**Algorithm 1:** AODV Routing Protocol1: **Input Parameters:**  N → The amount of deployed nodes (50) 2:    Coverage → Wireless communication range (25% of Network Area) 3: **Output Parameters:** Route → ‘S’ to ‘D’ Route 4: Initialize the variables 5: ‘S’: Corresponds to Source_Node; ‘D’: Respect to ‘Destination_Node 6: Nb-Add: Address of Nearby_Node; RREQ: Route request control message 7: RREP: Route reply control message; RP_Table: Maintain Reply Table 8: **Start Routing** 9:  Route = [] // Create an empty matrix to store Route Nodes 10: Route (1st Node) = S // Route 1st node is Source 11: **While** (D not founded) // Search Next Nodes in Route 12:  ‘S’ broadcast RREQ within Coverage Area 13:  **If** Nb-Add is ‘D’ **then** 14:   RREP acknowledge to the ‘S’ 15:   D founded 16:   Route (Next) = D // Consider D as a next node in the route 17:  **Else** 18:   This broadcasting process is continued until ‘D’ is not founded 19:   Route (Next) = Neighbour node with a minimum hop count 20:  **End—If** 21: **End—While** 22: RP_Table = Route // Store in the Table 23: **End—Function**

The first step is to pre-calculate the optimal route for some pre-existing gateways according to the routing schedule. If a new user requests data transmission, the new user must send a request to the nearest node on the network. This scenario is shown in Figure 4.

**Figure 5:**
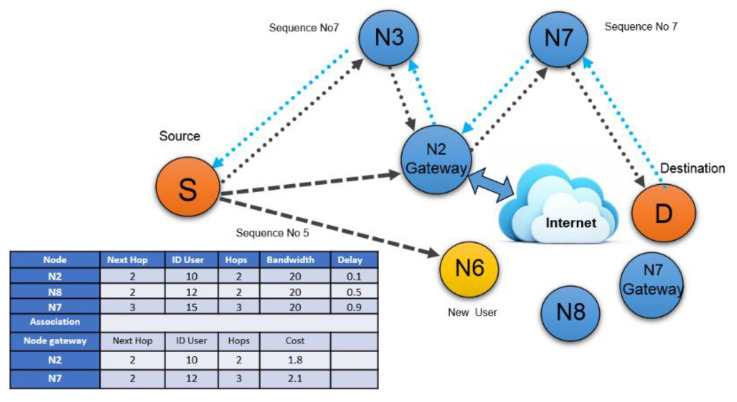
New user connection request using AODV.

Let us pretend that node (N6) is a brand new user in the network, as indicated by the yellow hue. Following receipt of this request, the AODV algorithm must consult the routing table (shown in Figure 5 under node and association) to determine the best pre-calculated path to the nearest gateway. Figure 5 illustrates this point (the next gateway two has a minimum hop number of two, so it will be used in this situation). After sending the specified route information to the new user (N6), a connection can be established. The network architecture, as well as network parameters, change when a new user joins. Mobile agents exchange this information with all nodes in the network while on the go.

A parallel procedure is triggered in the second stage of the routing process to assign a route to a new user, thus monitoring is focused on the new user’s traffic demands and optimizing bandwidth utilization and loading parameters at specified connections.

### 4.3. Artificial Bee Colony (ABC)

**Figure 6:**
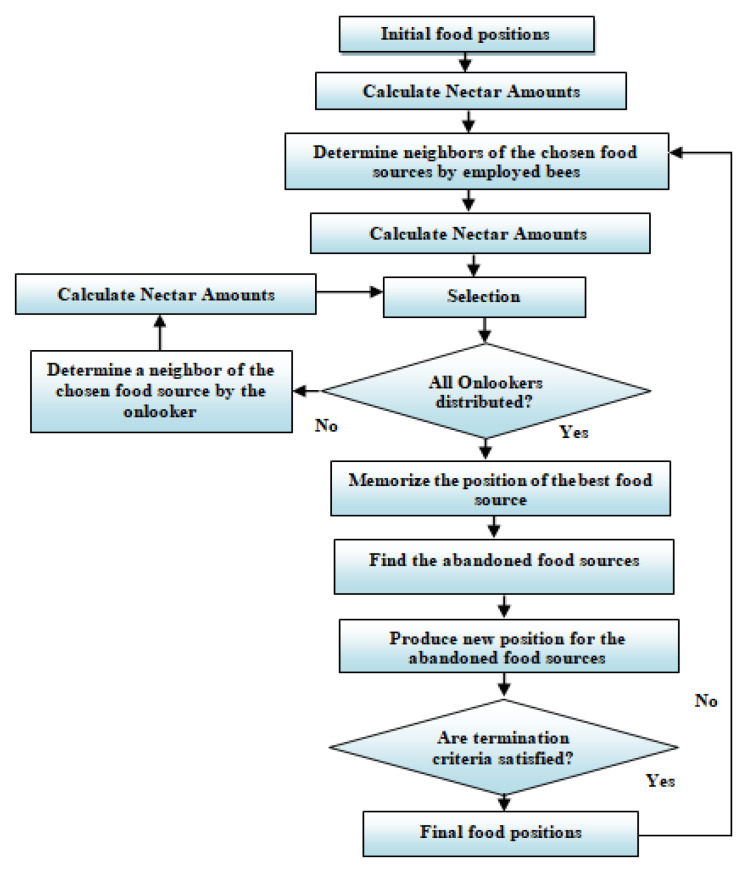
Working flow of ABC.

In this research, ABC is used for obtaining the best or most optimized node properties of the created route using the AODV protocol. ABC is a swarm-based metaheuristic algorithm used to solve combinatorial optimization challenges. The intelligent foraging behavior of bees acts as the inspiration of this algorithm which is particularly based on the model due to its foraging behavior of honeybee colonies. The aim of the ABC algorithm (Algorithm 2) is to differentiate the sensors nodes based on their basic features like coordinates, required time, and energy to transmit or receive the data packets using the fitness function. Here, total sensor node properties act as employee bees rather than the comparing parameter which is known as the threshold and acts as an onlooker bee. Based on the fitness of the bee, the ABC algorithm helps to differentiate the sensor nodes into two possible categories, such as communicating and non-communicating nodes. The workflow of ABC is shown in Figure 6.

To solve the complex problems in different domains, the foraging behavior of honeybees is considered. To establish communication between the bees, a waggle dance is required. That is composed of three pieces of information regarding the flower patch: (a) the direction in which it can go, (b) distance from the hive, and (c) its quality rating. To collect the knowledge of the outside environment separately, information is gathered through the waggle dance [32]. This dance enables the colony to compute the relative merit of multiple patches based on food quality as well as the energy amount to harvest it. The observer nodes observe the dance (energy consumed, delay, etc.) and based on that extract the food (apt node). The location of each food source (source and destination node) represents the optimized solution related to the problem. The working flow of ABC is shown in Figure 6.

**Figure 7:**
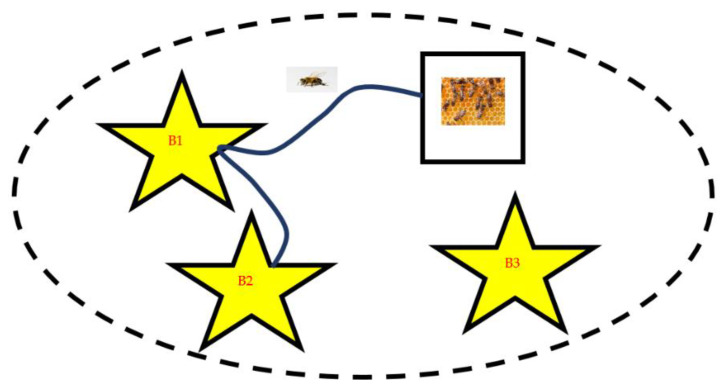
Scouting mechanism of ABC.

ABC is mainly composed of three kinds of bees: onlooker, forger, and scout bees. The onlooker bee’s role is to select a food source by just watching the waggle dance of the other bees. The role of forger bees is to constantly visit the food source to obtain nectar. Scout bees are those who conduct random searches to discover new sources of food [33]. The pictorial representation of the bees’ searching process of food is illustrated in Figure 7.

The main steps that are followed by ABC are listed below.

1. Initialize food sources as population size.
2. REPEAT:
  - Employed bees visit food sources and assess the amount of nectar based on their memories, and then return to the hive to perform a waggle dance at that location.
  - After the food source is exhausted, the onlooker bees start to randomly look for new food sources and memorize the best food source.
3. The process is repeated until the best food source is obtained.

Every time ABC visits a node (that is, the network topology or several nodes themselves), it computes the energy, delay, and packet drop rate. ABC includes an initialization process along with a search cycle process, iterating through the main search cycle until it finds the best suitable solution for the communicating node. After the arrival of the onlooker bees, the algorithm will strictly check to identify the source routing and the number of nodes present in the route performed by the AODV routing mechanism. If there is no source route, it will broadcast the packet and rescan the nodes’ properties to crosscheck whether it is used to transmit outgoing energy or incoming broadcast packets [34].

ABC with the AODV algorithm enhanced the routing process by selecting the best route between the source node and the destination node. The role of the scout bee is to measure the energy as well as the distance from one node to another node. The higher the distance is, the higher the transmission delay is which results in greater energy loss.

**Algorithm 2:** ABC Route Optimization1.  ** Input:** Final Route from S to D and Mobile Sensor Nodes Properties 2.  ** Output:** Optimized Mobile Sensor Nodes Properties 3.  **Start Properties Optimization** 4.  **To optimized the FR, ABC Algorithm is used** 5.  **Set up basic parameters of ABC:** Population of Bee ($P_{b}$)—Number of Sensor Nodes 6.   Final Route (FR)—Route from N_S_ to N_D_ 7.   Fitness Function: $F\left(f\right)=\{\begin{array}{c} 1;\;\mathrm{if}\;N_{\mathrm{PROP}}<{\mathrm{Threshold}}_{\mathrm{PROP}}\\ 0;\;\mathrm{Otherwise} \end{array}$ 8.   Where $N_{\mathrm{PROP}}$ are the properties of the node such as energy consumption, delay, etc. 9.   In the fitness function, $N_{\mathrm{PROP}}$: are properties of current sensor nodes which are in FR and ${\mathrm{Threshold}}_{\mathrm{PROP}}$ is the threshold properties of all communicating sensor nodes which are defined based on energy and distance 10. Calculate Length of Route in terms of R Length 11. **Set, Optimized Nodes Properties, ON_PROP_= []** 12. **For i in rang of R Length** 13.  E_BEE_ = FR (i) **=**
$N_{\mathrm{PROP}}$ // Current Bee from $P_{b}$ 14.  O_BEE_ = ${\mathrm{Threshold}}_{\mathrm{PROP}}$ // // Mean of all $P_{b}$ 15.  $F\left(f\right)=\;\mathrm{Fit}\;\mathrm{Fun}\;\left(E_{\mathrm{BEE}},{\;O}_{\mathrm{BEE}}\right)$ 16.  ON _PROP_ = ABC (F(f), FR (i)) 17. **End—For** 18. **Returns:** ON_PROP_ as an Optimized Mobile Sensor Nodes Properties 19. **End—Function**

### 4.4. Use of ML Techniques

In this research, the dual mechanism of an ML approach including ANN and SVM is used. This helps to provide double security to the network. SVM is used as a binary classifier that helps to select only supportable features of sensor nodes and these features help to train the network with more efficiency and more accurate data. Therefore, during the transmission of data packets, ANN can select better nodes in the network and the network efficiency will be better compared to the single AODV approach. Here, the supportable sensor nodes feature acts as an input of the ANN in terms of node properties like coordinates, required time, and energy to transmit or receive the data packets. Based on these properties, ANN can decide to segregate the nodes into two categories, such as communicating and non-communicating nodes within the route.

#### 4.4.1. Artificial Neural Network (ANN)

ANN is a technique of machine learning that is designed to work like a human brain. The working of ANN is similar to how a human brain works and memorizes from experience. ANN is a non-linear statistical model which processes input to discover a new pattern. The ANN consists of three layers as discussed below.

**Figure 8:**
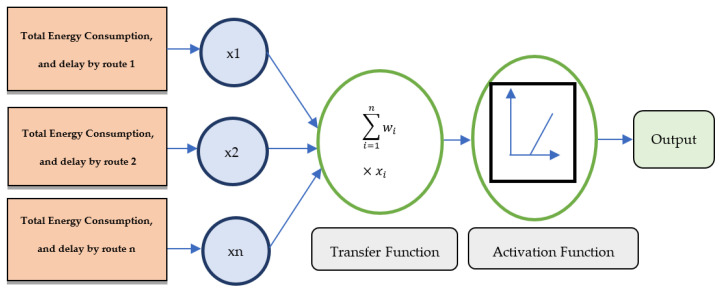
ANN Structure.

The process involves two attributes, namely energy consumption and delay, that are combined to create a single weight. This weight is then passed to one input node as illustrated in Figure 8.

Input layer: The input regarding the number of the optimized route is obtained based on the node’s properties, such as packet delay and energy consumed by nodes, which is provided as input information to the ANN.

Hidden Layer: This is positioned between the input and output of ANN. It can be single or multiple layers. Here, we used a single hidden layer. The main function of this layer is to process the input data to know the relationship between the attributes fed to the input layer.

Output layer: The resultant value after computation is obtained at this layer. The ANN computes the input values and provides the best route with minimum energy consumption and delay. The complete structure of ANN is shown in Figure 8.

Depending upon the optimized properties of nodes, the final output is obtained. The discrepancy between the input values and the output values is obtained in terms of error values. Based upon the error value, the weight of neurons in the hidden layer is adjusted and this process is known as backpropagation. Each neuron consists of two states which are 0 and 1, corresponding to deactivation and activation of the sigmoid function. Each neuron composed of weight $W_{\mathrm{ij}}$ corresponds to its interconnection. The case of $W_{\mathrm{ij}}$ = 0 represents the independence of the neuron, if $W_{\mathrm{ij}}$ = $W_{\mathrm{ji}}$ this shows that weights are symmetric. Every neuron behaves as an individual unit of an ANN structure with a non-linear transfer function given by Equation (1).

(1) $$f(x_{i})=Y_{i}=x_{i}$$

The neuron’s output is fed back to the other interconnected neurons by the linked weight of W = $W_{\mathrm{ij}}$. In the form of hardware, the weight corresponds to resistance, and weight in terms of resistance is given by Equation (2).

(2) $$W_{\mathrm{ij}}=\frac{1}{R_{\mathrm{ij}}}$$

The ANN includes two inputs: one is the external input and the other is the output of network neurons. Therefore, the total input to the neuron can be given by Equation (3).

(3) $$\underset{j\neq i}{\sum }\frac{x_{j}}{R_{\mathrm{ij}}}+{\mathrm{In}}_{i}$$

$R_{\mathrm{ij}}$ represents the resistance/interconnection of weight between neurons ‘i’ and ‘j’.

The output is obtained in the output layer of ANN in the form of two-state values. The output Y_j of output neuron ‘j’ provides values like Y_j^0 and Y_j^1, which represent values corresponding to 0 and 1, respectively. The output of input neuron (i) of x_i can be represented by Equation (4).

(4) $$\mathrm{If}\left(\underset{j\neq i}{\sum }\frac{x_{j}}{R_{\mathrm{ij}}}+{\mathrm{In}}_{i}<x_{j}\right)\left\{x_{j}=x_{i}^{0}\right\}$$

The state of the ANN can be identified by determining the energy function of the neurons. Mathematically, it can be represented by Equation (5).

(5) $$E=-\frac{1}{2}\overset{N}{\underset{i=1}{\sum }}\overset{N}{\underset{j=1}{\sum }}\frac{x_{i}x_{j}}{R_{\mathrm{ij}}}-\overset{N}{\underset{i=1}{\sum }}{\mathrm{In}}_{i}x_{i}$$

The energy is varied due to the alteration in the state-run of neurons (i) [13,35]. Therefore, the deviation in energy can be represented by Equation (6).

(6) $$\frac{\partial E}{\partial x_{i}}=-\overset{N}{\underset{j=1}{\sum }}\frac{x_{j}}{R_{\mathrm{ij}}}-\overset{N}{\underset{i=1}{\sum }}{\mathrm{In}}_{i}$$

**Figure 9:**
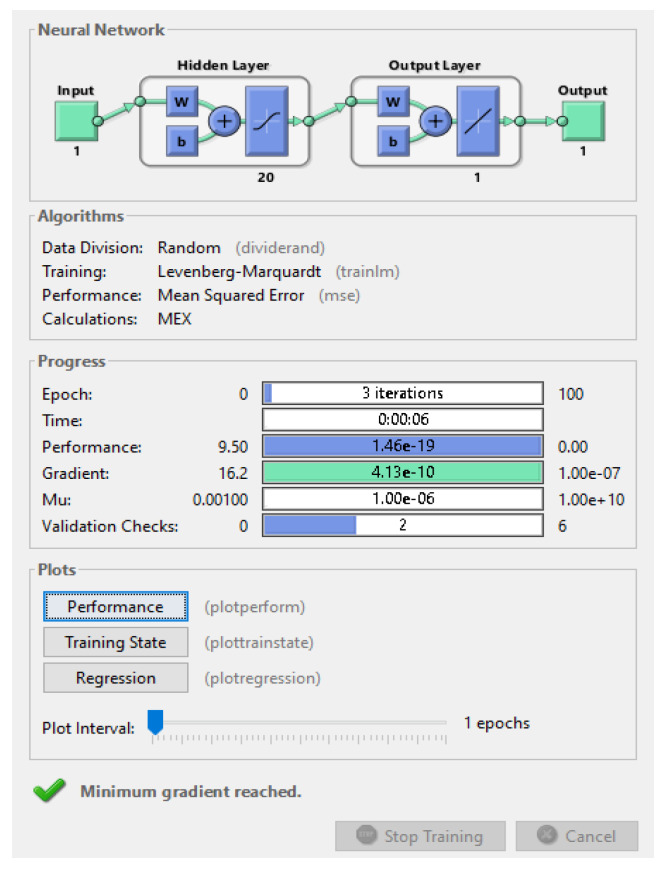
Trained ANN.

**Figure 10:**
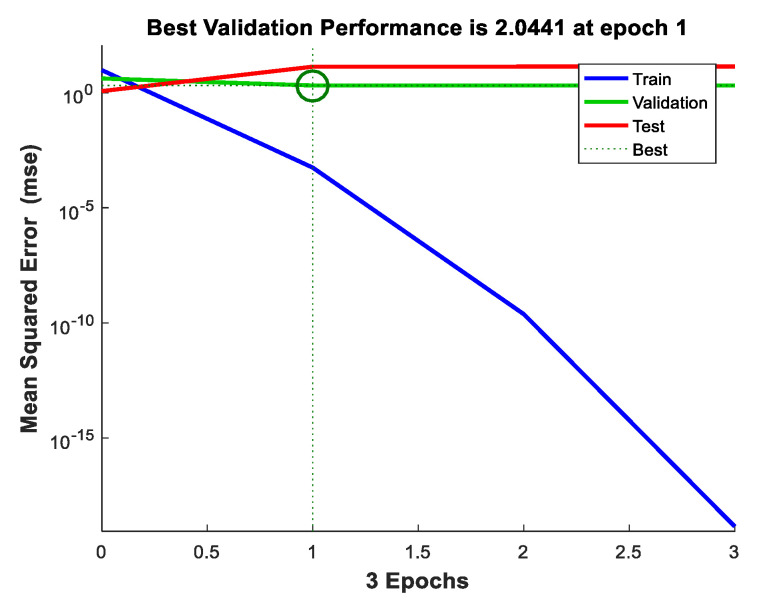
Mean square error (MSE).

The trained ANN structure and error graph generated during the training process are presented in Figure 9 and Figure 10, respectively.

For both normal and pathological nodes, the ANN is trained using an optimized node’s attributes. The attributes of nodes, such as the energy spent by nodes and the distance covered by nodes, are used as input parameters. If the result is not what is expected, the error is transmitted back to the hidden layer which adjusts the node attributes accordingly.

In this method, the network is trained with the least amount of error possible. The network in Figure 9 is made up of N interconnected neurons, as indicated by the arrow. These neurons individually update each neuron’s activation function. The mean square error is the error created by the ANN network during training, and it is depicted in Figure 10.

The MSE concerning epochs generated by the ANN algorithm is shown in Figure 10. The graph mainly consists of four different values as represented by different colors such as blue, green, red, and dotted lines, which represents the error values of the train, validation, test, and best-obtained solution, respectively. Here, the best-trained structure is obtained at the first epoch that carries an MSE of 2.0441. This is because RBF helps to reject the outermost properties of the nodes from the properties set and then SVM selects only support vectors as properties with minimum MSE [36]. The authors only run up to three epochs because the ANN targets are to fulfill the requirements in three epochs only and we achieved minimum MSE at three epochs, so there was no need for further epochs. We know that the training process of ANN is an iterative process and always depends upon the training parameters such as performances in terms of MSE, gradient, mutation, and validation check. From these parameters, if anyone fulfills the conditions of training then ANN automatically stops the training process. In Figure 10, it is clearly shown that the gradient parameter fulfills the training criteria of ANN, so ANN stops the training process. 

**Figure 11:**
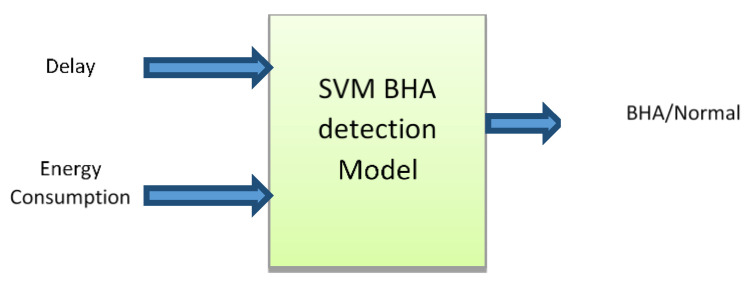
Block diagram of SVM.

The model does not face the problem of overfitting because the system achieves better MSE in just three epochs, which means the model avoids the overfitting problem. The step used to overcome the overfitting problem is used to reduce the complexity of the training structure of ANN, which is performed by considering multiple hidden layers in the trained ANN structure. According to Figure 11, a total of 20 hidden layers are used with 20 neurons that help to minimize the overfitting problem of ANN. Basically, ANN faces overfitting problems when lots of irrelevant node properties are considered as training data which negatively impacts the performance of the network, and to solve these types of problems, the concept of ABC as an optimization technique is used.

The aim of using ANN is to select the best route among the number of optimal routes obtained using AODV with the ABC approach. Now, the next step is to identify the black node if it is present in the selected route. To detect the node as a black hole node, the Support Vector Machine (SVM) approach is applied. The working of SVM is presented in Section 4.4.2.

#### 4.4.2. Support Vector Machine (SVM)

SVM is a supervised machine learning model known for its excellent performance in completing classification tasks with high-dimensional data [37,38,39,40,41]. Reference [13] stated that SVM is a better choice for the detection of a malicious node in the IDS system with high accuracy and minimum error. Therefore, the detection module considered in this study was developed by hybridizing learning models that are ANN and SVM. Here, SVM used RBF as a kernel function to select the best hyperparameters in this research work and we used the concept of RBF kernel function as a hyperparameter of SVM that is able to deal with polynomial data to create an optimal route from source to destination. A non-linear type kernel function is used in SVM to train the network and find the best route for data transmission in the network as a classifier or regression technique. Basically, it is a mapping function that is used to map the node data from one space (normal) to a new space (network with malicious nodes). The working of SVM is represented by the block diagram depicted in Figure 11.

The node properties of the selected route using the ANN approach are passed as inputs to the SVM model. The attributes of nodes such as delay and energy consumption are passed to train the module. The training data were collected using the ANN structure for both normal and attacker situations. Based on the energy and delay level, the decision was taken as a BHA node or normal node [42].

**Figure 12:**
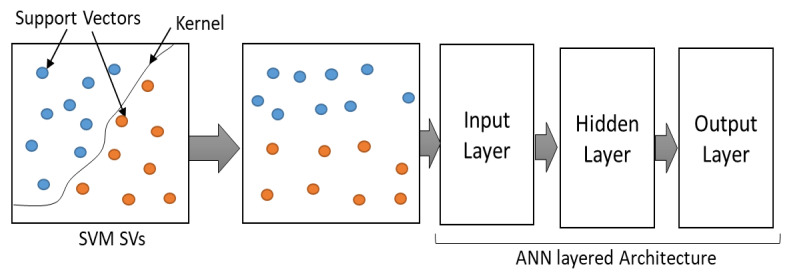
Information flow in ML model.

The algorithm (Algorithm 3) shows that the system is first trained using SVM to select the most relevant features to form the input feature set in terms of Support Vectors (SVs). These SVs represent the most appropriate feature set which is then passed to the ANN for training which assures the best possible classification. Hence, the output of SVM is used as the input of the ANN as illustrated in Figure 12 to differentiate between the normal and the malicious nodes.

**Algorithm 3:** SVM with ANN**Input Parameters:** FR → Final Route    ON_PROP_ → Optimized Nodes Properties as a Training Data (T),    C → In terms of communicative and non-communicating nodes, the target/category is **Output:** OR → Optimized Route for discovering a route from T_X_-Node to R_X_-Node and Malicious Nodes (M-Nodes) 1. **Start Training** 2. Set up the SVM training data. With RBF as the Kernel function, ONP is the total nodes optimised property. 3. **For I = 1** → **All Nodes** 4.  **If Property of Node (I) == Real** 5.   Defined the Cat as a category of training data 6.   Cat (1) = ON_PROP_ (I) 7.  **Else** 8.   Cat (2) = ON_PROP_ (I) 9.  **End—If** 10. **End—For** 11. Train_Structure = SVMTRAIN (T, Cat, Kernel function) 12. **OT= Train_Structure. Support-Vector** //To find out the training data for ANN 13. **Initialize the basic parameters of ANN**—Number of Epochs (E) // Iterations used by ANN         —Number of Neurons (N) // Used as a carrier in ANN         —Performance: MSE, Gradient, Mutation, and Validation         —Techniques: Levenberg Marquardt         —Data Division: Random 14. **For i = 1 → OT** 15.   **If T belongs to communicating nodes property** 16. Group (1) = Properties of training data according to the real nodes 17. **Else if T belongs to non-communicating nodes property** 18.  Group (2) = Properties of training data according to the non-real nodes 19. **Else** 20. Group (3) = Extra properties of training data 21. **End—If** 22. **End—For** 23. Initialized the ANN using Training data and Group 24. MANET-Net = Newff (T, Group, N) 25. Set the training parameters according to the requirements and train the system 26. VoIP = Train (MANET-Net, Training data, Group)  **Testing:** 27. Current Node = Properties of the current node in MANET -Net 28. Authentication = simulate (MANET-Net, Current Node) 29. **If Authentication = True** 30. Genuine Nodes do not consider as a malicious 31. **Else** 32. M-Nodes = Malicious Node 33. **End—If** 34. Create an Optimized Route, OR = FR (Genuine Nodes) 35. **Return:** OR as an Optimized Route (OR) and Malicious Nodes (M-Nodes) 36. **End—Function**

The following test cases were generated to investigate the intruder as shown in the Table 3.

## 5. Experiments and Analysis

### 5.1. Results

The results were computed in a MATLAB simulator that offers a simple platform for network simulation. The nodes were deployed in range of {N = 100, 200, 400, 600, 800, 1000, 2000}. The results were computed based on parameters such as throughput, PDR, and Delay (end-to-end). The obtained outcomes were examined using AODV along with ABC and the ANN approach [43]. Every performance parameter was performed with 100 iterations to achieve the best value. Comparative analyses were also performed to show the efficiency of the designed network. The performance of the designed black hole detection system was analyzed by evaluating parameters like PDR, throughput, and delay.

i. Packet Delivery Ratio (PDR)

This parameter is defined as the rate of data packets gained through the target node that are produced by the sources. Mathematically, PDR is represented by Equation (7).

(7) $$\mathrm{PDR}=\frac{\mathrm{Number}\;\mathrm{of}\;\mathrm{Packet}\;\mathrm{receive}}{\mathrm{total}\;\mathrm{Number}\;\mathrm{of}\;\mathrm{packet}}$$

PDR has an important role, and it shows the actual number of information carriers received by the receiver. The greater the value of PDR, the lesser amount of error inside the network.

ii. Throughput

The parameter throughput is described in MANET through the successful delivery of the message or delivery of packets throughout a communication network. Typically, throughput is estimated in bit/s or bps. Mathematically, it is given by Equation (8).

(8) $$\mathrm{Throughput}\left(\mathrm{Th}\right)=\frac{\mathrm{Number}\;\mathrm{ofpacket}\;\mathrm{recieve}}{\mathrm{Total}\;\mathrm{time}\;\mathrm{interval}}$$

iii. Delay

The delay parameter was defined as the ratio of the total length of time it takes packets to travel from the source to the destination node divided by the total number of data count packets. A total of 1000 packets started the data transmission. This is represented mathematically by Equation (9).

(9) $$\mathrm{Delay}=\frac{\mathrm{Delayed}\;\mathrm{packets}\;\mathrm{received}\;\mathrm{at}\;\mathrm{the}\;\mathrm{destination}}{\mathrm{total}\;\mathrm{count}\;\mathrm{of}\;\mathrm{packets}}$$

**Figure 13:**
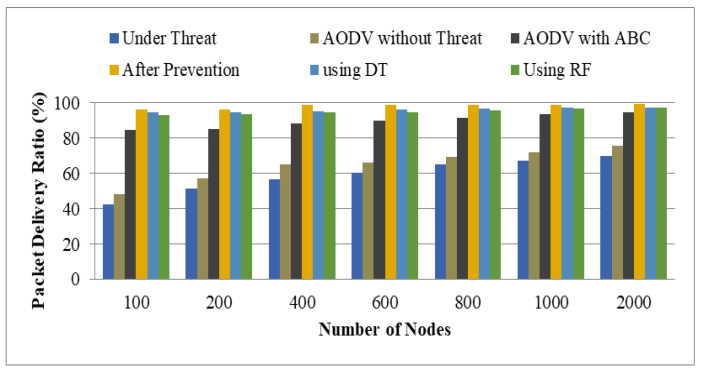
Packet delivery ratio.

Table 4 lists the computed values for the planned network based on the PDR measure, and Figure 13 depicts the graph. PDR is the ratio of the total number of packets received to the total number of packets transmitted. The graph shows four different values.

- Threat to Ad-hoc On-Demand Distance Vector (AODV)
- AODV with Threat
- AODV with Artificial Bee Colony (ABC)
- Artificial Neural Network (ANN) and Support Vector Machine (SVM) with AODV (after prevention)
- Using Decision Tree (DT)
- Using Random Forest (RF)

As per the given graph shown in Figure 13, it is clear that the proposed approach (modified AODV) using ABC and ANN with the SVM method performed well. The values were analyzed concerning the number of nodes as indicated on the x-axis. To show the enhancement comparison, other considered scenarios were presented. The average value analyzed with AODV under threat, AODV without threat, AODV with ABC, after prevention, using Decision Tree (DT), and using Random Forest (RF) was observed as 59.07, 64.94, 89.67, 97.96, 96.08, and 95.12, respectively. Therefore, the highest PDR was examined for the proposed work. This is because of the dual ML algorithm that improves the attack detection rate and hence improves the Quality of Service (QoS) parameter.

**Figure 14:**
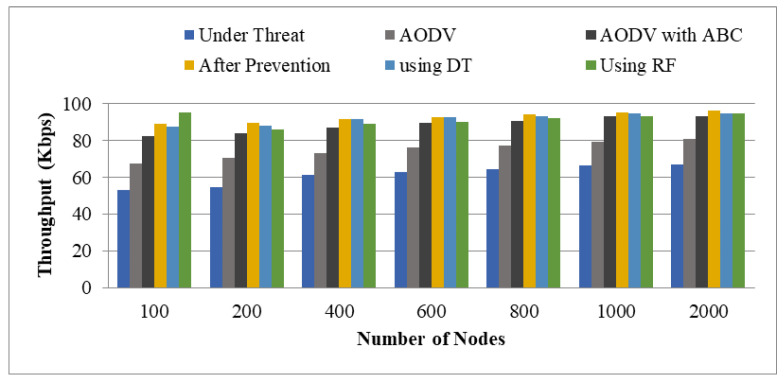
Throughput.

Throughput represents the transmission information of packets in a network. The analyzed values for four different scenarios with different numbers of nodes 100, 200, 400, 600, 800, 1000, and 2000 are listed in Table 5. Figure 14 depicts a graphical depiction of throughput.

The suggested job has a larger throughput than the other five cases: AODV under Threat, AODV with Threat, AODV with ABC, utilizing DT, and using RF. When the BHA occurs in the network, the system’s performance suffers. To address this issue, AODV is utilized as a routing protocol, combining the benefits of ABC with a dual scenario of SVM and ANN. The path is improved and the attacker node is recognized using ABC in conjunction with ANN and SVM. As a result, throughput rose and 61.38, 74.98, 88.71, 92.78, 91.82, and 91.62 were the average values examined for AODV under Threat, AODV, AODV with ABC, after prevention, using DT, and using RF classifiers, respectively.

Delay represents the total time taken by the packets to arrive at the destination node (D) via the source node(s). The analyzed values of delay are shown in Table 6.

**Figure 15:**
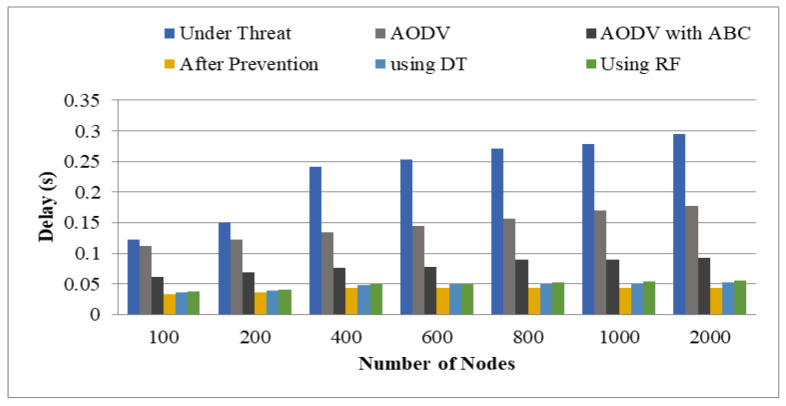
Delay.

Figure 15 depicts the delay values studied for various methodologies. The blue color, red color, green color, violet color, sky blue color, and the orange color reflects the values of delay under threat, AODV, AODV with ABC, after prevention from BHA, using DT, and using RF, respectively. The average delay utilizing AODV under danger, AODV without threat, AODV with ABC, after prevention, using DT, and RF was 0.2304 s, 0.145 s, 0.079 s, and 0.04 s, 0.046 s, and 0.048 s, respectively.

The following hypothesis was established and tested using a t-test in order to validate the outcome before and after the application of the suggested work set.

- Hypothesis

**H0.**  There is no significant difference in the performance of the routing protocol, before or after the preventions structure.

**H1.**  Otherwise.

A *t*-test is a type of statistical test used to find out a significant difference between the means of two values analyzed before and after applying the optimization approach to detect malicious nodes. The analyzed values before and after the prevention algorithm are mentioned in Table 7.

The average values analyzed before and after the prevention algorithm are summarized in Table 7, which proved the H0 hypothesis to be wrong.

### 5.2. Discussion

As most of the work performed by researchers to detect black hole attacks is performed using DT and RF techniques, we considered DT and RF techniques to compare our proposed hybrid classification SVM with the ANN approach to show the efficacy of the work. From the abovementioned work, it was observed that an improvement of 1.96% in PDR was obtained against DT and 2.99% against RF approach. Similarly, improvement in terms of throughput of 1.05% and 1.27% were observed against DT and RF approaches. An improvement of 13.04% and 16.67% occurred while evaluating delay using the proposed approach in comparison to DT and RF approaches, respectively.

### 5.3. Comparative Analysis

The studied performance was compared to prior work presented by Ali Zardariet al. [40] and Gupta et al. [13] to prove the efficiency of the constructed secure MANET against BHA utilizing the offered hybrid classification technique. The proposed work’s average PDR, throughput (Kbps), and delay (ms) were compared to the average PDR, throughput, and delay of existing works, as shown in Table 8.

With [13,34], a comparative study of the planned work with previous work was carried out. In comparison to previous studies, it was discovered that the created hybrid AI strategy (SVM plus ANN) performed better. This is because the authors utilized a classic technique known as the CDS (Connected Discriminant Set) approach in [34], in which data transmission was done based on only one feature of nodes, namely the energy spent by nodes. The suggested study, on the other hand, employed an intelligent technique in which ANN and SVM are trained utilizing two separate features of nodes, such as energy consumption and data transmission latency. According to previous research [34], the risks of packet drop rise as each node retains a data packet for an extended period. To address this issue, we developed a BHA detection model that uses Artificial Intelligence (AI) to identify network intrusions automatically, with average PDR, throughput, and latency of 97.96 percent, 92.78 kbps, and 0.04 s, respectively.

The reliability factor technique was employed by the authors in [13], in which the reliability factor of the nodes was determined, and if the computed value was high, the data were sent; otherwise, the data were rechecked for malicious behavior using the FRREQ message. If a node is malicious, it will respond to the FRREQ and be labeled as such. The procedure is time-consuming and yields a low PDR rate. As a result, to improve the work we used an optimization strategy using hybrid classification approaches to improve AODV routing and were able to achieve higher network performance in terms of PDR, throughput, and latency. The results comparison is described later.

The comparison of PDR against the existing work performed by [13,40] is depicted in Table 7. There is an improvement of 1.29% and 3.21%, respectively, against the work in [13,34]. This is due to the appropriate selection of nodes while creating routes among source and destination nodes. The existing approach [34] worked on the energy consumption of nodes properties whereas in [13] the nodes were identified using a reliability score. In the proposed work, three nodes properties were considered for the identification of BHA nodes and therefore performed better compared to existing approaches.

The comparative analysis for throughput analyzed against [34] work is also depicted in Table 7. The percentage increase in the throughput was calculated and the improvement of $\left(\frac{92.78-79.08}{79.08}\right)$ × 100 = 17.32% was examined compared to the work in [34].

In comparison to prior work by [13,34], the average delay measured for the proposed work was 0.087 ms and 0.035 ms, respectively. The enhancement is shown in Table 7. To know the percentage enhancement against existing work, the percentage decrease was calculated. $\left(\frac{0.087-0.04}{0.087}\right)$ × 100 = 54.02% from [40] and $\left(\frac{0.035-0.04}{0.035}\right)$ × 100= 14.29% against [13] improvement in the proposed work was obtained.

## 6. Conclusions

All the networks used in combination with IoT are susceptible to challenges of security and facing these issues is one of the noticed points for developers in an IoT system. If the network has faced such problems during the design and data transmission, other systems are necessary to create security. In the previous years, various strategies have been proposed to take care of this issue. Because of the dynamic idea of these systems and their capacity to transmit through remote methods, the security of information and data are increasingly significant in these systems. The main motive of this paper was to distinguish the black hole node and divert the route through the protected node utilizing an altered AODV routing protocol with SVM and ANN in the IoT-MANET. The novelty of this research work helps to achieve better performance during the data packet transmission observed after identifying the node as the black hole in the route and is performed dependent on the energy consumption, delay in transmission of data, and the positioning of nodes in the system. As per the obtained outcome of these experiments, the presented AODV with ABC, ANN, and SVM approach performed well with an average of PDR, throughput, and delay of 97.96%, 92.78 Kbps, and 0.04 s, respectively. Therefore, we conclude that the combination of the SVM with ANN for optimized nodes properties using ABC is a beneficial step compared to the other traditional algorithms like Decision Tree and Random Forest. In the future, this work can be extended to minimize delays with improved PDR and throughout. The work can also be extended to other fields like rescue operations in the military and personal area networking.

## Figures and Tables

**Table 1:** Node (normal and malicious) behavior.

Malicious	Destination Sequence Number	Hop Count	Packet Drop	Threat	Threat Type
No	High	High	No	No	No Threat
Yes	No	No	Yes	Yes	BHA
Yes	No	High	Yes	Yes	BHA
Yes	High	No	Yes	Yes	BHA
Yes	High	High	Yes	Yes	Gray hole

**Table 2:** Simulation Parameter.

**Running Time**	0.2 ms to 1 ms
**The Number of Deployed Nodes**	100 to 200
**Malicious Nodes Count**	1
**Simulation Environment**	1000 × 1000

**Table 3:** Test cases generation

**Test Case Count**	**Attributes**		
	$\mathrm{Nda}=\frac{\mathrm{node}}{\mathrm{deployment}\;\mathrm{area}}$	Judgment Parameter	Analysis Parameter
7	[0.1–2]	Throughput, Delay	Energy Consumption

**Table 4:** Packet delivery ratio.

Number of Nodes	Under Threat	AODV without Threat	AODV with ABC	After Prevention	Using DT	Using RF
100	42.5	48.52	84.55	96.45	94.52	93.26
200	51.25	57.41	85.22	96.47	94.87	93.48
400	56.75	65.22	88.13	98.75	95.26	94.75
600	60.66	66.27	89.72	98.76	96.27	94.68
800	65.37	69.47	91.67	98.74	96.99	95.82
1000	67.2	72.24	93.74	99.17	97.12	96.78
2000	69.77	75.47	94.72	99.38	97.59	97.13

**Table 5:** Throughput.

Number of Nodes	Under Threat	AODV	AODV with ABC	After Prevention	Using DT	Using RF
100	53.02	67.8	82.7	89.2	87.56	95.26
200	54.83	70.67	84.22	89.8	87.89	86.29
400	61.3	73.13	87.08	91.79	91.78	89.15
600	62.9	76.17	89.51	92.92	92.67	90.36
800	64.29	77.09	90.87	94.3	93.26	92.45
1000	66.34	79.2	93.3	95.22	94.75	93.17
2000	67.02	80.82	93.33	96.23	94.89	94.68

**Table 6:** Average Delay(s).

Number of Nodes	Under Threat	AODV	AODV with ABC	After Prevention	Using DT	Using RF
100	0.123	0.112	0.062	0.033	0.0363	0.0375
200	0.1504	0.123	0.0694	0.036	0.0392	0.0412
400	0.2416	0.135	0.0767	0.043	0.0481	0.0496
600	0.2527	0.145	0.0781	0.0431	0.0495	0.0499
800	0.2718	0.156	0.892	0.0433	0.0501	0.0521
1000	0.2781	0.17	0.904	0.0437	0.0512	0.0534
2000	0.2956	0.178	0.922	0.0439	0.0523	0.0554

**Table 7:** Statistical test values.

PDR (%)		Throughput (Kbps)		Delay (s)	
Before Prevention	Proposed	Before Prevention	Proposed	Before Prevention	Proposed
59.07	97.96	61.38	92.78	0.2304	0.04

**Table 8:** Comparative parametric value.

PDR (%)			Throughput (Kbps)		Delay (s)		
Proposed	[34]	[13]	Proposed	[34]	Proposed	[34]	[13]
97.96	96.94	95.14	92.78	79.08	0.04	0.087	0.035

## Data Availability

The data used in the current study are available from the corresponding author on reasonable request.

## References

[1] Alnumay W., Ghosh U., Chatterjee P. (2019). A Trust-Based Predictive Model for Mobile Ad Hoc Network in Internet of Things. Sensors.

[2] Ghayvat H., Mukhopadhyay S., Gui X., Suryadevara N. (2015). WSN- and IOT-Based Smart Homes and Their Extension to Smart Buildings. Sensors.

[3] Masek P., Masek J., Frantik P., Fujdiak R., Ometov A., Hosek J., Andreev S., Mlynek P., Misurec J. (2016). A Harmonized Perspective on Transportation Management in Smart Cities: The Novel IoT-Driven Environment for Road Traffic Modeling. Sensors.

[4] Deng Y.-Y., Chen C.-L., Tsaur W.-J., Tang Y.-W., Chen J.-H. (2017). Internet of Things (IoT) Based Design of a Secure and Lightweight Body Area Network (BAN) Healthcare System. Sensors.

[5] Tamilselvan L., Sankaranarayanan V. (2008). Prevention of Co-operative Black Hole Attack in MANET. J. Netw..

[6] Kang B.-S., Ko I.-Y. (2010). Effective Route Maintenance and Restoration Schemes in Mobile Ad Hoc Networks. Sensors.

[7] Himral L., Vig V., Chand N. (2011). Preventing aodv routing protocol from black hole attack. Int. J. Eng. Sci. Technol. (IJEST).

[8] Panigrahi R., Borah S., Bhoi A.K., Ijaz M.F., Pramanik M., Kumar Y., Jhaveri R.H. (2021). A Consolidated Decision Tree-Based Intrusion Detection System for Binary and Multiclass Imbalanced Datasets. Mathematics.

[9] Papadimitratos P., Haas Z. Secure routing for mobile ad hoc networks. Proceedings of the SCS Communication Networks and Distributed Systems Modeling and Simulation Conference (CNDS 2002).

[10] Cai R.J., Li X.J., Chong P.H.J. (2019). An Evolutionary Self-Cooperative Trust Scheme Against Routing Disruptions in MANETs. IEEE Trans. Mob. Comput..

[11] Djahel S., Nait-Abdesselam F., Zhang Z. (2010). Mitigating Packet Dropping Problem in Mobile Ad Hoc Networks: Proposals and Challenges. IEEE Commun. Surv. Tutor..

[12] Gaur L., Singh G., Solanki A., Jhanjhi N.Z., Bhatia U., Sharma S., Verma S., Kavita, Petrović N., Ijaz M.F. (2021). Disposition of Youth in Predicting Sustainable Development Goals Using the Neuro-fuzzy and Random Forest Algorithms. Hum.-Cent. Comput. Inf. Sci..

[13] Gupta P., Goel P., Varshney P., Tyagi N. (2019). Reliability factor-based AODV protocol: Prevention of black hole attack in MANET. Smart Innovations in Communication and Computational Sciences.

[14] Mohanapriya M., Krishnamurthi I. (2014). Modified DSR protocol for detection and removal of selective black hole attack in MANET. Comput. Electr. Eng..

[15] Gurung S., Chauhan S. (2020). A survey of black-hole attack mitigation techniques in MANET: Merits, drawbacks, and suitability. Wirel. Netw..

[16] Seyedi B., Fotohi R. (2020). NIASHPT: A novel intelligent agent-based strategy using hello packet table (HPT) function for trust Internet of Things. J. Supercomput..

[17] Thebiga M., SujiPramila R. (2020). A New Mathematical and Correlation Coefficient Based Approach to Recognize and to Obstruct the Black Hole Attacks in Manets Using DSR Routing. Wirel. Pers. Commun..

[18] Lee C., Jeong T. (2011). FRCA: A Fuzzy Relevance-Based Cluster Head Selection Algorithm for Wireless Mobile Ad-Hoc Sensor Networks. Sensors.

[19] Gurung S., Chauhan S. (2018). A dynamic threshold based approach for mitigating black-hole attack in MANET. Wirel. Netw..

[20] Mohammadani K., Memon K.A., Memon I., Hussaini N.N., Fazal H. (2020). Preamble time-division multiple access fixed slot assignment protocol for secure mobile ad hoc networks. Int. J. Distrib. Sens. Netw..

[21] El-Semary M., Diab H. (2019). BP-AODV: Blackhole Protected AODV Routing Protocol for MANETs Based on Chaotic Map. IEEE Access.

[22] Arunmozhi S.A., Venkataramani Y. (2012). Blackhole attack detection and performance improvement in mobile ad-hoc network. Inf. Secur. J. Glob. Perspect..

[23] Shahabi S., Ghazvini M., Bakhtiarian M. (2015). A modified algorithm to improve security and performance of AODV protocol against black hole attack. Wirel. Netw..

[24] Baadache A., Belmehdi A. (2012). Fighting against packet dropping misbehavior in multi-hop wireless ad hoc networks. J. Netw. Comput. Appl..

[25] Kumari S.V., Paramasivan B. Ant-based defense mechanism for selective forwarding attack in MANET. Proceedings of the 2015 31st IEEE International Conference on Data Engineering Workshops.

[26] Gurung S., Chauhan S. (2018). A novel approach for mitigating gray hole attack in MANET. Wirel. Netw..

[27] Keerthika V., Malarvizhi N. (2019). Mitigate Black Hole Attack Using Hybrid Bee Optimized Weighted Trust with 2-Opt AODV in MANET. Wirel. Pers. Commun..

[28] Merlin R.T., Ravi R. (2019). Novel Trust Based Energy Aware Routing Mechanism for Mitigation of Black Hole Attacks in MANET. Wirel. Pers. Commun..

[29] Rezaei R., Medadian M., Darvishi M. Provide a way to deal with attacks on black holes in wireless networks case: The behavior of nodes. Proceedings of the National Conference on Computer Engineering and Information Technology Management.

[30] Yasin A., Abu Zant M. (2018). Detecting and Isolating Black-Hole Attacks in MANET Using Timer Based Baited Technique. Wirel. Commun. Mob. Comput..

[31] Sood M., Verma S., Panchal V.K., Kavita (2019). Optimal Path Planning Using Swarm Intelligence Based Hybrid Techniques. J. Comput. Theor. Nanosci..

[32] Kumar M., Mukherjee P., Verma K., Verma S., Rawat D.B. (2021). Improved Deep Convolutional Neural Network based Malicious Node Detection and Energy-Efficient Data Transmission in Wireless Sensor Networks. IEEE Trans. Netw. Sci. Eng..

[33] Ghosh G., Kavita, Anand D., Verma S., Rawat  D.B., Shafi J., Marszałek Z., Woźniak M. (2021). Secure Surveillance Systems Using Partial-Regeneration-Based Non-Dominated Optimization and 5D-Chaotic Map. Symmetry.

[34] Zardari Z.A., He J., Zhu N., Mohammadani K.H., Pathan M.S., Hussain M.I., Memon M.Q. (2019). A Dual Attack Detection Technique to Identify Black and Gray Hole Attacks Using an Intrusion Detection System and a Connected Dominating Set in MANETs. Future Internet.

[35] Lv Z., Qiao L., Verma S., Kavita (2021). AI-enabled IoT-Edge Data Analytics for Connected Living. ACM Trans. Internet Technol..

[36] Hussain A., Nazir S., Khan F., Nkenyereye L., Ullah A., Khan S., Verma S., Kavita (2021). A Resource Efficient hybrid Proxy Mobile IPv6 extension for Next Generation IoT Networks. IEEE Internet Things J..

[37] Mukkamala S., Sung A.H. Detecting denial of service attacks using support vector machines. Proceedings of the 2003 12th IEEE International Conference on Fuzzy Systems, FUZZ ’03.

[38] Rani S., Koundal D., Kavita, Ijaz M.F., Elhoseny M., Alghamdi M.I. (2021). An Optimized Framework for WSN Routing in the Context of Industry 4.0. Sensors.

[39] Singh P., Singh R.P., Singh Y., Shafi J., Ijaz M.F. (2021). An Enhanced Naked Mole Rat Algorithm for Optimal Cross-Layer Solution for Wireless Underground Sensor Networks. Mathematics.

[40] Padhi D.K., Padhy N., Bhoi A.K., Shafi J., Ijaz M.F. (2021). A Fusion Framework for Forecasting Financial Market Direction Using Enhanced Ensemble Models and Technical Indicators. Mathematics.

[41] Shams E.A., Rizaner A. (2018). A novel support vector machine-based intrusion detection system for mobile ad hoc networks. Wirel. Netw..

[42] Rani P., Kavita, Verma S., Nguyen G.N. (2020). Mitigation of Black Hole and Gray Hole Attack Using Swarm Inspired Algorithm with Artificial Neural Network. IEEE Access.

[43] Datta D., Dhull K., Verma S. (2020). UAV Environment in FANET: An Overview. Applications of Cloud Computing.

